# An update on post-translational modifications of hydroxyproline-rich glycoproteins: toward a model highlighting their contribution to plant cell wall architecture

**DOI:** 10.3389/fpls.2014.00395

**Published:** 2014-08-14

**Authors:** May Hijazi, Silvia M. Velasquez, Elisabeth Jamet, José M. Estevez, Cécile Albenne

**Affiliations:** ^1^Laboratoire de Recherche en Sciences Végétales, Université de Toulouse, UPS, UMR 5546Castanet-Tolosan, France; ^2^CNRS, UMR 5546Castanet-Tolosan, France; ^3^Facultad de Ciencias Exactas y Naturales, Instituto de Fisiología, Biología Molecular y Neurociencias (IFIBYNE-CONICET), Universidad de Buenos AiresBuenos Aires, Argentina

**Keywords:** arabinogalactan protein, extensin, hydroxyproline, *O*-glycosylation, proline-rich protein

## Abstract

Plant cell walls are composite structures mainly composed of polysaccharides, also containing a large set of proteins involved in diverse functions such as growth, environmental sensing, signaling, and defense. Research on cell wall proteins (CWPs) is a challenging field since present knowledge of their role into the structure and function of cell walls is very incomplete. Among CWPs, hydroxyproline (Hyp)-rich *O*-glycoproteins (HRGPs) were classified into three categories: (i) moderately glycosylated extensins (EXTs) able to form covalent scaffolds; (ii) hyperglycosylated arabinogalactan proteins (AGPs); and (iii) Hyp/proline (Pro)-Rich proteins (H/PRPs) that may be non-, weakly- or highly-glycosylated. In this review, we provide a description of the main features of their post-translational modifications (PTMs), biosynthesis, structure, and function. We propose a new model integrating HRGPs and their partners in cell walls. Altogether, they could form a continuous glyco-network with non-cellulosic polysaccharides via covalent bonds or non-covalent interactions, thus strongly contributing to cell wall architecture.

## Introduction

Plant cell walls are composite structures mainly composed of polysaccharides, namely cellulose, hemicelluloses and pectins, containing also a large set of proteins involved in the cell dynamics through diverse functions such as growth, environmental sensing, signaling, and defense (Fry, 2004). Research on cell wall components emerged in the nineteen sixties (Lamport and Northcote, 1960; Rees and Wight, 1969) and is still a very active field with continuous advances on the nature, structure and functions of polysaccharides (Carpita and Gibeaut, 1993; Willats et al., 2006; Scheller and Ulvskov, 2010) and of proteins (Rose and Lee, 2010; Albenne et al., 2013). However, the question of how these components are connected to make a functional matrix is still a matter of debate (Keegstra et al., 1973; Park and Cosgrove, 2012; Wang et al., 2012).

Among cell wall proteins (CWPs), hydroxyproline (Hyp)-rich *O*-glycoproteins (HRGPs) are complex macromolecules with various structures and functions. Identified several decades ago, HRGPs were classified into three categories: (i) moderately glycosylated extensins (EXTs); (ii) hyperglycosylated arabinogalactan proteins (AGPs); and (iii) Hyp/Pro-rich proteins (H/PRPs) that may be non-, weakly- or highly-glycosylated. Each HRGP sub-family is characterized by repetitive consensus sequences which determine the way they are glycosylated according to the so-called Hyp-*O*-glycosylation code (Kieliszewski, 2001; Tan et al., 2004; Estevez et al., 2006). From a functional point of view, HRGPs are also very diverse. AGPs are implicated in a variety of physiological processes including cell expansion, reproductive development, embryogenesis, signaling, and defense (Seifert and Roberts, 2007). EXTs are mostly described as structural proteins able to form covalent scaffolds (Qi et al., 1995; Brady et al., 1996; Cannon et al., 2008; Velasquez et al., 2012). Finally, H/PRPs are the less documented HRGPs and little is known about their structure and function. They seem to be associated to development and defense against biotic and abiotic stresses (Bradley et al., 1992; Bernhardt and Tierney, 2000; Battaglia et al., 2007). Hybrid and chimeric HRGPs also exist, enlarging the diversity of this superfamily. As previously defined, hybrid HRGPs are composed of HRGP modules from different families, and chimeric HRGPs are composed of one or more HRGP modules within a non-HRGP protein (Showalter et al., 2010). An expert bioinformatics analysis of the *Arabidopsis thaliana* genome identified 166 HRGPs classified in 85 AGPs, 59 EXTs, 18 H/PRPs, and 4 AGP/EXT hybrid proteins (Showalter et al., 2010). Besides, related to HRGPs but not classified in any of its three sub-families, some allergen proteins containing Hyp residues substituted by arabinogalactans (AGs) were identified in *Artemisia vulgaris* and *Ambrosia artemisiifolia* (Léonard et al., 2005, 2010).

Despite the great interest that plant biologist have had in HRGPs for more than 50 years, many questions about their mode of action in cell walls are still unanswered and HRGP research is still very challenging. In this review, we provide an update on (i) their post-translational modifications (PTMs) which consist in Pro-hydroxylation and *O*-glycosylation on serine (Ser) and Hyp residues and (ii) their roles in cell walls. We also focus on new insights into HRGP supramolecular assembly and propose a model including most recent data on covalent and non-covalent networks connecting HRGPs and polysaccharides.

## Extensins (EXTs)

### EXTs as structural molecules in plant cell walls

EXTs are modular, highly repetitive HRGPs showing similar features as collagen that contain Tyr cross-linking motifs. Unlike collagen, EXTs also undergo plant specific post-translational *O*-glycosylation on Ser-(Hyp)_*n* ≥ 2_ motifs. EXTs are represented in the *A. thaliana* genome by 59 members, some are classical EXTs while others are EXT-like chimeras and hybrid-EXTs that also contain other domains. Despite the high number of proteins with EXT domains in plant cell walls (Lamport et al., 2011), we know little about their exact functions and how this protein diversity is coordinated during plant development. There are several reasons that may explain our current lack of understanding of the EXT biology: (i) a high similarity in their protein sequences that make their characterization at the molecular level very difficult; (ii) the highly repetitive nature of their sequences since they are modular proteins, large in size and with complex chemical structures that carry several PTMs. Consequently, the biochemical characterization of a single EXT protein is still today very challenging; (iii) large number of EXTs and EXTs-related proteins encoded in known plant genomes; and (iv) several EXT genes are expressed at the same time in the same plant tissues (see Genevestigator database, https://www.genevestigator.com). In addition, most of the available EXT mutants analyzed until now show no clear phenotype. Few exceptions are the mutants *atext3* (embryo lethal), *atext6, 7, 10, 12* (shorter root hairs) and *lrx1, 2* (root hair morphogenesis) that showed clear phenotypes (see Table 1).

**Table 1:** **Examples of EXTs and EXT-related proteins characterized in the last years**.

**Protein/Gene name**	**Tissue or sub-cellular localization**	**Assumed function /Phenotype of mutants**	**References**
**EXTs**			
AtEXT1 (At1g76930)	Roots and Inflorescences	Cell wall formation/Induction in response to mechanical wounding, pathogen infection, senescence and at abscission zones, and treatment with hormones (methyl jasmonate, salicylic acid, auxin, brassinosteroids)	Merkouropoulos and Shirsat, 2003
AtEXT3 (At1g21310)	Embryo	Cell wall formation/Embryo-lethal mutant. Incomplete cross wall assembly	Hall and Cannon, 2002; Cannon et al., 2008
AtEXT6 (At2g24980)	Root hairs	Cell wall formation/Short root hair	Velasquez et al., 2011
AtEXT7 (At4g08400)			
AtEXT10 (At5g06640)			
AtEXT11 (At5g49080)			
AtEXT12 (At4g13390)			
AtMOP10 (At5g05500)	Root hairs	Cell wall formation/Short root hair	Velasquez et al., 2011
AtEXT-LIKE (At4g26750)	Root hairs	Cell wall formation/Short root hair	Velasquez et al., 2011
SlEXT1	Trichoblasts	-/Induced by ethylene	Bucher et al., 2002
BnExtA	External and internal phloem of the main stem	-/Greatest expression in regions where a maximum tensile stress is exerted	Shirsat et al., 1996
NtEXT1.4	Stems, Roots and Carpels	-/Cells under mechanical stress: emergence of lateral roots, junction stem/petiole, fusion of carpels. Induction by mechanical stress in roots and stems	Hirsinger et al., 1999; Salvà and Jamet, 2001
NsEXT1.2A	Stems and Roots	-/Expression in the root transition zone, in stem inner and outer phloem and in cortical cells at the stem/petiole junction. Induced by wounding	Guzzardi et al., 2004
**LRXs**			
AtLRR-EXT (At4g29240)	Root hairs	Cell wall formation /Short root hair	Velasquez et al., 2011
AtLRX1 (At1g12040)	Root hairs	Cell wall formation/Morphogenesis of root hair	Baumberger et al., 2001
AtLRX2 (At1g62440)	Root hairs	*atlrx2* acts synergistically with *atlrx1*. *atlrx1/atlrx2* show osmophilic aggregates and local disintegration of the cell wall	Baumberger et al., 2003
VcISG (Inversion-Specific Glycoprotein)	Extracellular matrix	–	Ertl et al., 1992
ZmPex1/ZmPex2/SlPEx (Pollen extensin-like)	Callose portion of the pollen tube cell wall	–	Rubinstein et al., 1995; Stratford et al., 2001

Dc, Daucus carota; Dca, Dianthus caryophyllus; La, Lupines albus; Ns, Nicotiana sylvestris; Nt, N. tabacum; Sl, Solanum lycopersicon; Vc, Volvox carteri; Zm, Zea mays.

### PTMs of EXTs and the enzymes involved

Structural *O*-glycoproteins containing EXT domains that are ultimately secreted into plant cell walls are first shaped by several and complex PTMs that include: (i) signal peptide processing (in the ER), (ii) hydroxylation of Pro into Hyp residues, (iii) *O*-glycosylation on Hyp and Ser residues (in the ER-Golgi apparatus) and finally, (iv) Tyr cross-linking to promote the formation of a covalent network (in the cell wall). In the last few years, great progress has been made in our knowledge of the molecular players that act on the EXT biosynthetic pathway with the identification of several enzymes involved in their PTMs (summarized in Table A1). Hydroxylation of peptidyl-Pro is catalyzed by prolyl 4-hydroxylases (P4Hs) providing reactive hydroxyl groups for further *O*-glycosylation. Plant P4Hs are membrane-bound enzymes that belong to a family of 2-oxoglutarate-dependent dioxygenases (Hieta and Myllyharju, 2002; Koski et al., 2007, 2009). Partial *in vitro* and *in vivo* characterization of plant P4Hs (see Table A1) has been carried out in several plant model systems (Hieta and Myllyharju, 2002; Tiainen et al., 2005; Yuasa et al., 2005; Keskiaho et al., 2007; Vlad et al., 2007, 2010; Asif et al., 2009; Velasquez et al., 2011, 2012, in revision; Parsons et al., 2013). Most P4Hs are able to hydroxylate with different affinities several types of substrates containing collagen-like, polyproline EXT-type as well as AGP-like sequences. On the other hand, structural information on plant P4Hs is scarce since only one P4H from *Chlamydomonas reinhardtii* (CrP4H1) has been crystallized (Koski et al., 2007, 2009) and few P4Hs were characterized *in vivo* (Velasquez et al., in revision). Recent evidence showed that in *A. thaliana*, P4H5 forms homo-/hetero-dimers with P4H2 and P4H13 in the Golgi, suggesting the existence of P4H complexes required for proper Pro hydroxylation. It is plausible that more than one type of P4H complex would be formed in the ER-Golgi compartment, and in the case of the hetero-complexes, the presence of specific P4Hs (e.g., AtP4H5) may be required either for the correct recruitment or the scaffolding of the other P4Hs (e.g., AtP4H2) (Velasquez et al., in revision).

Hydroxylated EXTs are usually *O*-glycosylated with chains of up to four linear Ara residues on each Hyp (Velasquez et al., 2011; Ogawa-Ohnishi et al., 2013). The usual arabinoside structure linked to each Hyp unit is composed of β-L-Ara*f*-(1,2)-β-L-Ara*f*-(1,2)-β-L-Ara*f*-(1,3)-α-L-*t*Ara*f*. A fifth arabinose unit was reported in some tissues (Lamport, 1973). Specifically, three groups of arabinosyltransferases (AraTs) HPAT1-HPAT3 (GT8 CAZy family), RRA1-RRA3 (GT77 family), and XEG113 (GT77 family) have recently been implicated in the sequential addition of the innermost three L-Ara residues (Egelund et al., 2007; Gille et al., 2009; Velasquez et al., 2011; Ogawa-Ohnishi et al., 2013). The AraT that would transfer the fourth (1,3)-α-L-Ara*f* moiety was identified very recently as *E*xtensin *A*rabinose *D*eficient transferase (ExAD) within the GT47 family (Petersen et al. *in preparation*). In addition, one novel peptidyl-Ser galactosyltransferase named as SGT1 has been reported to add a single α-Gal*p* residue to each Ser residue in Ser-(Hyp)_4_ motifs of EXTs and it would belong to a new family of CAZy (Saito et al., 2014). Glycosylated EXTs are cross-linked, at least *in vitro*, by putative type-III peroxidases (PERs) at the Tyr residues (Schnabelrauch et al., 1996; Jackson et al., 2001; Price et al., 2003) forming *intra*- and *inter*-EXT linkages (Cannon et al., 2008; Lamport et al., 2011). Thus, EXTs are able to form a three-dimensional glycoprotein network that possibly interacts with other cell wall components like pectins (Nuñez et al., 2009; Dick-Perez et al., 2011). Although the *in vivo* molecular mechanism of the covalent cross-link is unknown, there is evidence of PER-catalyzed oxidative coupling of Tyr residues *in vitro* that mediates the insolubilization of the proteins (Schnabelrauch et al., 1996; Jackson et al., 2001; Price et al., 2003). Recently, six apoplastic type-III PERs were identified as putative candidates for the cross-linking of EXTs specifically in the root hairs of *A. thaliana* (Velasquez et al., in revision). Structural proteins with polyproline sequences like collagen can also be Tyr-cross-linked by the action of a PER not only *in vitro* but also *in vivo* (Edens et al., 2001) suggesting that EXTs and collagen, as extracellular building blocks, would share structural features and functions.

#### Root hair as models to study EXT functions and related GTs

Root hairs have been used as a single-cell model to study cell wall biosynthesis in general and specifically EXTs during tip-growth (Park et al., 2011; Velasquez et al., 2011). Mutants deficient in the synthesis of a single wall polymer specifically in the root hair are generally impaired in growth because their cell wall structure is severely compromised (Diet et al., 2006; Cavalier et al., 2008; Ringli, 2010; Park et al., 2011; Pena et al., 2012; Velasquez et al., 2012). In this framework of interconnected wall polymers (Cosgrove, 2005; Dick-Perez et al., 2011), cross-linked EXTs have a key role during cell expansion and growth (Cannon et al., 2008; Ringli, 2010; Lamport et al., 2011; Velasquez et al., 2011). EXT domains seem to be important during polarized cell expansion since several EXT-related mutants have shorter root hairs such as classical *ext6*, *ext7*, *ext10* and *ext12* (Velasquez et al., 2011, 2012) and *lrx1* and *lrx2* mutants (Baumberger et al., 2001, 2003; Ringli, 2010).

#### Impact of O-glycosylation on EXT function

It is accepted that *O*-glycans increase HRGP solubility, resistance to proteolytic degradation and thermal stability (Kieliszewski et al., 1989; Ferris et al., 2001; Shpak et al., 2001; Kieliszewski et al., 2011; Lamport et al., 2011). Most of the mutants that correspond to glycosyltransferases (GTs) known to glycosylate EXTs (Table A1) have been related to root hair drastic phenotypes, highlighting that even minor changes in the *O*-glycosylation status of EXTs affect EXT function during polarized cell expansion (Velasquez et al., in revision). In addition, it was found that both *O*-glycosylation types present in EXTs (Hyp-*O*-arabinosylation and Ser-*O*-galactosylation) are required and have additive effects for correct EXT function in root hair growth (Velasquez et al., in revision). The known roles of EXTs in cell wall assembly, cell shape and growth raise the question about the function of each individual EXT molecule (Hall and Cannon, 2002; Cannon et al., 2008; Velasquez et al., 2011). Some examples of already characterized EXT or EXT-related genes are presented in Table 1. Recently, it was reported that EXTs can form, at least *in vitro*, a tridimensional covalent network through Tyr-linkages mediated by EXT PERs between individual EXT molecules and also via self-recognition and alignment of hydrophilic *O*-glycosylated Ser-(Hyp)_3−4_ repeats and hydrophobic peptide-cross-linking modules (Cannon et al., 2008). Thus, the ordered EXT monomer assembly in plant cell walls would involve a zipper-like endwise association via cross-linking at the ends of the molecules (Kieliszewski et al., 2011; Lamport et al., 2011). Recently, molecular dynamics and homology modeling experiments suggested that classical EXTs would be able to form a putative triple helix structure by lateral staggered alignment (Cannon et al., 2008) and Tyr cross-linking, similar to that present in collagen (Velasquez et al., in revision). It is also proposed that EXTs interact with pectins by a simple acid-base reaction forming a supramolecular ionic structure in the nascent cell wall (Valentin et al., 2010), which would serve as a template for further cell wall deposition (Cannon et al., 2008; Lamport et al., 2011). In addition, covalent EXT-pectin cross-links were also suggested (Qi et al., 1995; Nuñez et al., 2009). However, it is unclear how EXT monomers are secreted and assembled into the glyco-network and how EXT-pectin interactions are controlled in a coordinated way during new cell wall formation. In addition, pectin methyl esterases de-esterify galacturonic acid residues in homogalaturonans and liberate acidic charges for ionic interactions (Micheli, 2001) with positively-charged domains in molecules like EXTs.

## Arabinogalactan proteins (AGPs)

Many articles reporting the state of the art concerning AGP structure, function and biosynthesis have been published recently (Seifert and Roberts, 2007; Ellis et al., 2010; Tan et al., 2012; Lamport and Várnai, 2013; Nguema-Ona et al., 2013; Knoch et al., 2014). AGPs are HRGPs containing a high proportion of sugars, up to 90%. They are characterized by repetitive X(Pro)_*n*_ motifs in which X is mostly alanine (Ala) or Ser. In this review, we focus on specific aspects concerning (i) the characterization of their *O*-glycan moiety and (ii) their interactions with cell wall polysaccharides.

### Structure of *O*-glycans of AGPs

**Figure 1:**
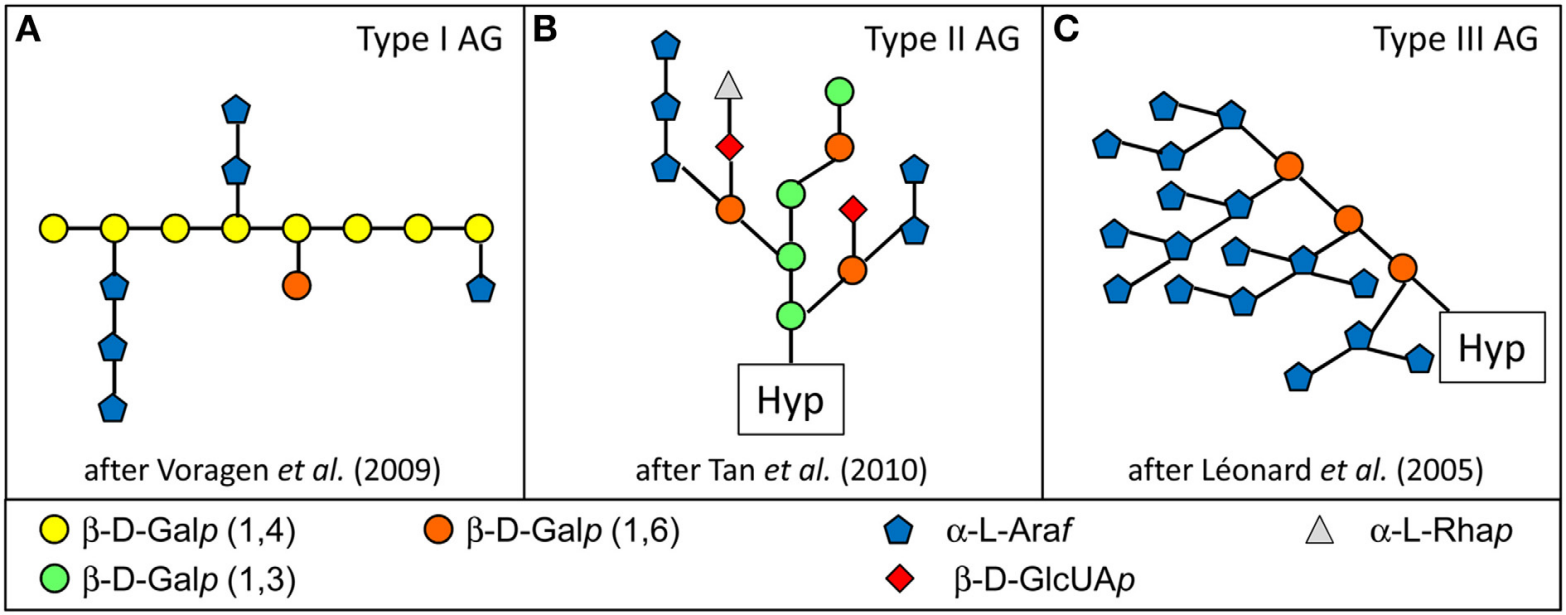
**The three main types of AGs**. One of the main differences between these AG types consist in the type of linkages between Gal residues of the main chain: β-1,4 in type I AG **(A)**; β-1,3 and β-1,6 in type II AG **(B)**; β-1,6 in type III AG **(C)**. These differences have been highlighted on the figure by using different colors for Gal residues. Other differences are described in the text.

A remarkable work performed on proteins deriving from synthetic genes and produced in cell suspension cultures has allowed to characterize AGP *O*-glycans covalently linked to [Ser(Hyp)]_*n*_ and [Ala(Hyp)]_*n*_ motifs (Tan et al., 2010). It has been possible to precisely define the structure of type II AGs by combining monosaccharide and linkage analyses to mass spectrometry and NMR. An example of type II AG is given in Figure 1B. Type II AGs contain a β-D-Gal*p* backbone formed by a succession of three β-1,3 linked D-Gal*p* interrupted by a β-1,6 linkage causing a reverse turn. Gal residues of side chains can be substituted with α-L-Ara*f*, α-L-Rha*p* or Me-Glc*p*A (Tan et al., 2010; Tryfona et al., 2012). The chelation of Ca^2+^ ions could occur at the level of GlcA residues located in close proximity. It should be noted that different variants of this basic structure exist, for example β-1,6 side chains can vary in length from 1 over 20 Gal residues (Tryfona et al., 2012). Type II AGs react positively with the β-glucosyl and β-galactosyl Yariv reagents, but not with the α-glucosyl and α-galactosyl Yariv reagents (Kitazawa et al., 2013). The β-galactosyl Yariv reagent has been shown to recognize the β-1,3-Gal main chains of type II AGs. They are different from previously described type I AGs which constitute lateral branches of RGI (Voragen et al., 2009). Type I AGs are formed by a linear chain of β-D-Gal*p* (1,4) with lateral chains of α-L-Ara*f* (1,5 attached to Gal O-3) and β-D-Gal*p* (attached to Gal-O-6) (Figure 1A). Type II AGs also differ from type III AGs found on allergens like the *Artemisia vulgaris* Art v 1 (Léonard et al., 2005). The structure of type III AGs has been determined by combining the results of linkage analysis, NMR and enzymatic degradation. They are formed by a short linear chain of β-D-Gal*p* (1,6). They only contain Gal and Ara residues, and they have large branched Ara chains. The linkage analysis indicates the presence of terminal Ara*f*, 5-Ara*f*, 3,5-Ara*f*, 2,5-Ara*f*, 2,3,5-Ara*f* and 3,6-Gal*p*. This Hyp *O*-glycan was shown to consist of Hyp_1_Gal_3_Ara_5−28_ series by MALDI-TOF MS. Type III AGs react with the β-glucosyl Yariv reagent suggesting that Art v 1 is an AGP. As for type II AGs, type III AGs probably exist in various forms and only a consensus model can be proposed (Figure 1C). Another kind of type III AGs has been later described for Amb a 4, an allergen of *Ambrosia artemisiifolia* (Léonard et al., 2010). It differs from the that of Art v 1 by the presence of different Hyp_1_Gal_1_Ara_5−20_series with a lower amount of Gal, the presence of more α-Ara*f* (1,5) and less α-Ara*f* (1,3).

The existence of different types of AGs linked to AGPs (types II and III) raises the questions of (i) the role of the amino acid sequence and (ii) the presence of different types of GTs in plants to ensure the appropriate *O*-glycosylation of HRGPs (Léonard et al., 2005).

### Interactions of AGPs with polysaccharides

The question of how AGPs are connected to other cell wall components and the involvement of their carbohydrate moieties in the interactions is of great importance, but still poorly documented. It has been assumed that AGPs could act as covalent cross-linkers in polysaccharide networks. Several lines of evidence suggested associations between AGPs and pectins. More than 40 years ago, it was hypothesized that Rha residues on type II AG side chains could be attached to RGI (Keegstra et al., 1973). Since then, several studies have reported the existence of strong associations between AGPs and pectins from different plant tissues, including grape (Pellerin et al., 1995), carrot (Immerzeel et al., 2006) or sugar beet (McKenna et al., 2006). Pectins were shown to co-localize with AGPs in pollen tubes (Li et al., 1995; Jauh and Lord, 1996; Mollet et al., 2002). Besides, enzymatic treatment of cell wall fractions with pectin-degrading enzymes allowed for an increased release of AGPs (Immerzeel et al., 2006; Lamport et al., 2006). One study also suggested the existence of AGP/xylan complexes (Kwan and Morvan, 1995). However, all these AGP/polysaccharide complex analyses were either indirect or achieved on preparations containing a mixture of AGPs, thus preventing a detailed characterization of the interactions. The first in depth structural study of an AGP polysaccharide complex involving pure AGP was only recently reported (Tan et al., 2013). It was shown that two isoforms of a purified *A. thaliana* AGP, At3g45230, are covalently attached to pectins and hemicelluloses. Linkages have been demonstrated between: (i) RGI/homogalacturonan and the Rha residue in the AGP type II AG domain and (ii) arabinoxylan and either a Rha residue of RGI or an Ara residue in the type II AG domain. A model was proposed for this complex called Arabinoxylan Pectin Arabinogalactan Protein1 (APAP1). The *apap1* mutant showed an increased extractability of pectin and xylan, suggesting a structural role for APAP1 (Tan et al., 2013). However, since APAP1 was isolated from suspension culture media, it could correspond to a simplified structure with pectin and xylan domains smaller than expected in plant cell walls. Larger APAP1 complexes may exist in cell walls, but their extraction is undoubtedly the bottleneck preventing their characterization. Large AGP/pectin/xylan complexes should also be found in other plants, corroborating all prior studies indirectly suggesting their existence (Tan et al., 2013).

Present knowledge on AGP/polysaccharide interactions indicates that some AGPs may serve as cross-linker in cell walls and act as polysaccharide plasticizers as previously assumed (Lamport, 2001; Lamport et al., 2006). Chimeric proteins containing AGP domains were also suggested to interact with polysaccharides. In particular, SOS5 (SALT-OVERLY SENSITIVE 5), a Fasciclin-AGP, was assumed to interact with pectins, thus mediating mucilage adherence (Griffiths et al., 2014). SOS5 interacting partners were not identified. Further efforts will be necessary to highlight the contribution of AGPs to cell wall architecture and to give more insight into its molecular basis.

## Hyp/Pro-rich proteins

Like EXTs and AGPs, H/PRPs belong to the HRGP superfamily and some of them are chimeric proteins. As mentioned above, little is known about the *O*-glycosylation of H/PRPs and their interactions with polysaccharides. With regard to *O*-glycosylation, information is only available for H/PRPs having X(Pro/Hyp)_*n* ≥ 2_X motifs. This type of domain can be associated with a short N-terminal AGP domain, a histidine (His)-stretch and a C-terminal PAC (Proline-rich protein and AGP, containing Cys) domain like in the *A. thaliana* AtAGP31 (Liu and Mehdy, 2007; Hijazi et al., 2012). Up to now, twelve such proteins have been identified in *A. thaliana*, *Daucus carota*, *Gossypium hirsutum*, *Nicotiana alata*, *N. tabacum*, *Phaseolus vulgaris*, *Capsicum annuum* and *Petunia hybrida* (Hijazi et al., 2014).

### Structure of the *O*-glycans of H/PRPs of the AtAGP31 type

*O*-glycosylated amino acid motifs of the H/PRP domain of AtAGP31 have been characterized by mass spectrometry: Lys(Ala/Ser)HypVal, Lys(Pro/Hyp)(Hyp/Pro)(Thr/Val), Thr(Pro/Hyp)(Hyp/Pro)Val, and Tyr(Pro/Hyp)(Hyp/Pro)Thr (Hijazi et al., 2012). The monosaccharide linked to Hyp is an hexose which is most probably a Gal based on the monosaccharide analysis of the purified protein (53.2% Gal, 39.5% Ara, 2.2% Xyl, 1.9% Fuc, 1.8% Glc, 1.3% Man, 0.3% GlcUA). It should be noted that this global analysis includes *O*-glycans linked to the AGP domain of AtAGP31 and *N*-glycans linked to its PAC domain. The *O*-glycan linked to the H/PRP domain of AtAGP31 is not recognized by the β-D-glucosyl Yariv reagent, but it interacts with the Peanut Agglutinin (PNA), a lectin having a high affinity for Gal residues (Hijazi et al., 2012). It was called Gal/Ara-rich motif (Hijazi et al., 2012). *Nicotiana alata* NaPRP4 shares the same type of H/PRP domain and a PAC domain with AtAGP31 (Sommer-Knudsen et al., 1996). The predominant monosaccharide of this *O*-glycoprotein is Gal (83%) wheareas Ara, GlcNac, Man, Xyl are in minor amounts (7, 4, 4, 1% respectively). The linkage analysis has shown the presence of terminal Ara*f* (6%), terminal Gal*p* (48%), 1,3-Gal*p* (4%), 1,6-Gal*p* (14%), 1,3,6 Gal*p* (25%), 1,2-Man*p* (1%) and Xyl*p* (1%). Altogether, H/PRPs with X(Pro/Hyp)_*n* ≥ 2_X motifs are *O*-glycosylated with Gal-Ara-rich glycans which seems to be slightly different from the previously described type I, II and III AGs. Further characterization, especially by NMR will be required to fully describe these structures.

### Interactions of H/PRPs with polysaccharides

H/PRPs are assumed to be cross-linked in cell walls, but direct evidence is still lacking (Bradley et al., 1992; Brisson et al., 1994; Frueauf et al., 2000). Nothing is known about the possible roles of *O*-glycosylations. AtAGP31was recently proposed to be involved in non-covalent interactions networks (Hijazi et al., 2014). Consistently and unlike HRGPs which are covalently insolubilized in cell walls, AtAGP31 is easily extracted from cell walls of etiolated hypocotyls (Hijazi et al., 2012). It should be noted that NaPRP4 is not insolubilized in cell walls as well (Sommer-Knudsen et al., 1996). AtAGP31 was shown to interact *in vitro* with RGI type I AG branches through its PAC domain and with methyl-esterified polygalacturonic acid, probably through its His-stretch. Protein/protein interactions were also assumed for AtAGP31, with (i) self-recognition between its PAC domain and its H/PRP domain *O*-glycans, and (ii) interaction with cell wall lectins. It was proposed that the AtAGP31 multi-domain organization results in complex supra-molecular scaffolds with different cell wall components, thus contributing to the strengthening of cell walls of quickly growing organs like etiolated hypocotyls. Such non-covalent networks have not been described before for HRGPs. A similar behavior may exist for proteins sharing features with AtAGP31 (Hijazi et al., 2014). However, as mentioned above, except NaPRP4 whose glycosylation has been characterized (Sommer-Knudsen et al., 1996), these proteins were not described at the molecular level and their interactions with cell wall polysaccharides were not investigated. TTS-1 and TTS-2 (Transmitting Tissue-Specific) from *N. tabacum*, and DcAGP1 from *D. carota* were shown to display an ellipsoidal shape and to self-assemble into higher-order structures using microscopy techniques (Baldwin et al., 2000, 2001; Wu et al., 2001). Interestingly, the deglycosylation of TTS disrupts its ability to aggregate, suggesting a regulation of self-association by its level of *O*-glycosylation (Wu et al., 1995). Self-assembly in a head-to-tail fashion through interactions between the *O*-glycans of H/PRP domain and the PAC domain can be proposed for proteins like TTS and DcAGP1, similarly to AtAGP31.

## Concluding remarks and future developments

**Figure 2:**
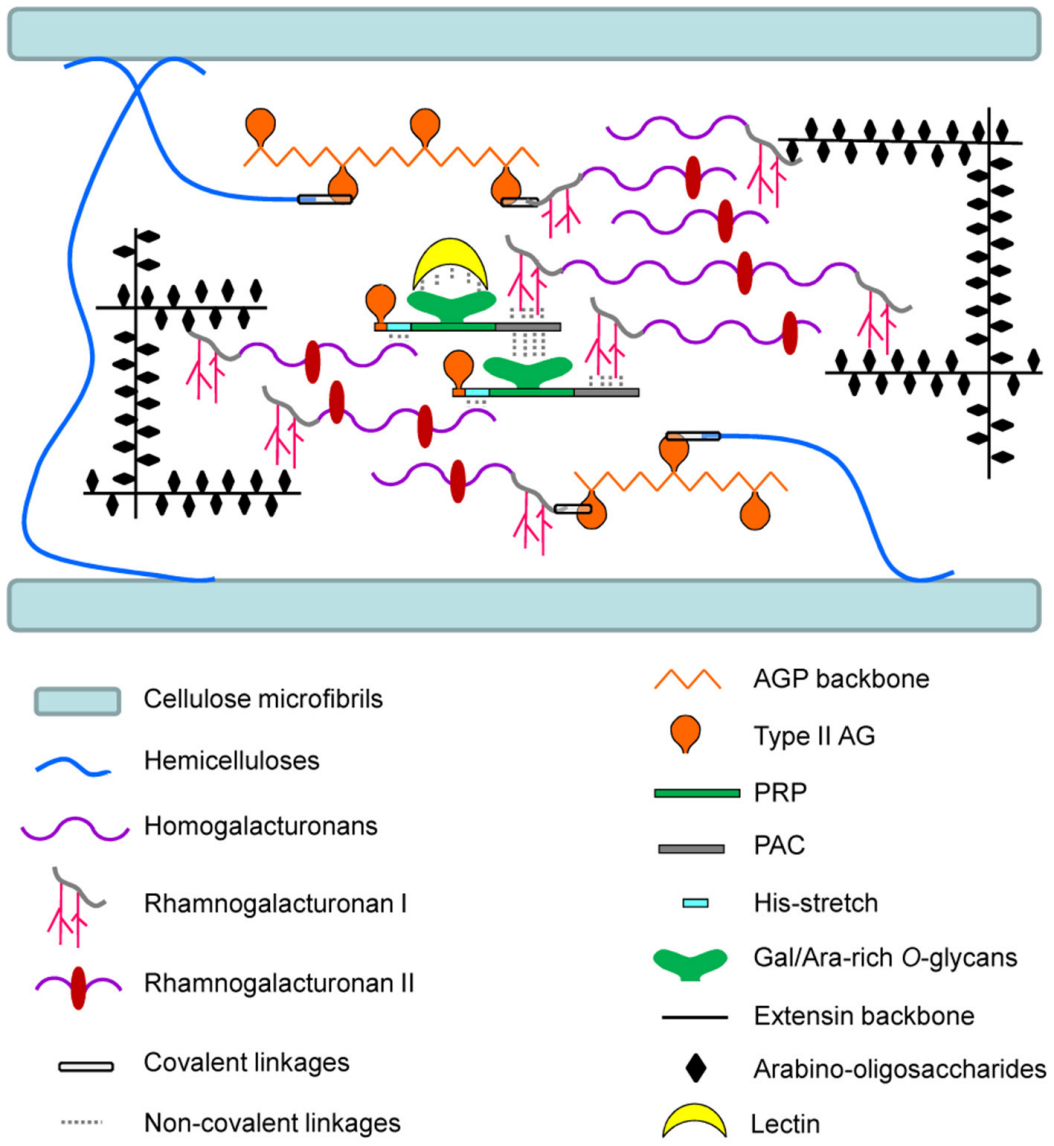
**Schematic representation of interactions between HRGPs and cell wall polysaccharides *in muro***. This model proposes an overview of the interactions assumed or demonstrated between HRGPs and polysaccharides according to most relevant publications in this field. For clarity, the model does not represent the whole complexity of the polysaccharide networks. AGPs are represented with covalent linkages with pectins and hemicelluloses, as proposed by Tan et al. (2013) for the so-called APAP1 complex. EXTs are drawn attached covalently with pectins as proposed by Qi et al. (1995). Finally, non-covalent networks between chimeric HRGPs and polysaccharides are represented according to Hijazi et al. (2014) for AtAGP31. Lectins assumed to bind to Gal/Ara-rich *O*-glycans of AtAGP31 are also integrated into the model.

In this review, we have focused on some structural features of HRGP *O*-glycans and we have highlighted their possible interactions *in muro* through covalent glycosidic linkages or non-covalent interactions. As proposed in the model shown in Figure 2, HRGPs could serve as cross-linkers in cell walls, connecting non-cellulosic polysaccharides, thus forming a continuous network. Large covalent complexes connecting AGP, hemicelluloses and pectins, as proposed in APAP1, are represented (Tan et al., 2013). However, the relevance of such covalent complexes in cell walls need to be confirmed. EXTs appear to form covalent linkages with pectins as reported (Qi et al., 1995; Nuñez et al., 2009). The precise moieties involved in these linkages have not been identified so far. Finally, chimeric HRGPs with H/PRP and PAC domains like AtAGP31 may form non-covalent networks with a set of cell wall components, including polysaccharides and lectins (Hijazi et al., 2014). It can be speculated that these protein/polysaccharide networks contribute to the cell wall architecture, by reinforcing the polysaccharide scaffold and by controlling its porosity. A recent high-resolution solid-state NMR study elucidating the 3D-architecture of the polysaccharides and proteins *in muro* revealed that the structural proteins in the primary cell wall are separated from the polysaccharides by more than one nanometer (Wang et al., 2012). This corroborates the assumption that *O*-glycans acts as spacers between HRGP backbones and cell wall polysaccharides.

These new features render even more complex the cell wall architecture. Plant cell walls contain a variety of complex macromolecules, possibly interconnected, resulting from a sophisticated metabolism. A tremendous set of carbohydrate active enzymes is required to achieve (i) polysaccharide synthesis and assembly, (ii) protein glycosylation, and (iii) possible polysaccharide/protein linkages. Non-cellulosic polymer synthesis occurs in the Golgi (Mohnen, 2008; Brown et al., 2011), and HRGP synthesis starts in the ER and continues in the Golgi (Basu et al., 2013; Knoch et al., 2014). An important issue is now to determine in which sub-cellular compartment covalent HRGP/polysaccharide complexes are formed and by which mechanism. Is there a code for establishing these links or are they occurring randomly? Which enzymes are involved? Answering these questions constitutes a real challenge toward a better understanding of cell wall biosynthesis and architecture. Further studies will also be necessary to elucidate the molecular basis of HRGP functions in cell walls and their involvement in physiological processes like cell plate formation or root hair cell expansion (Cannon et al., 2008; Velasquez et al., 2011).

### Conflict of interest statement

The authors declare that the research was conducted in the absence of any commercial or financial relationships that could be construed as a potential conflict of interest.

## References

[B1] Albenne C. Canut H. Jamet E. (2013). Plant cell wall proteomics: the leadership of *Arabidopsis thaliana*. Front. Plant Sci. 4:111 10.3389/fpls.2013.00111 23641247 PMC3640192

[B2] Asif M. H. Trivedi P. K. Misra P. Nath P. (2009). Prolyl-4-hydroxylase (AtP4H1) mediates and mimics low oxygen response in *Arabidopsis thaliana*. Funct. Integr. Genomics 9, 525–535 10.1007/s10142-009-0118-y 19277739

[B3] Baldwin T. C. Domingo C. Schindler T. Seetharaman G. Stacey N. Roberts K. (2001). DcAGP1, a secreted arabinogalactan protein, is related to a family of basic proline-rich proteins. Plant Mol. Biol. 45, 421–435 10.1023/A:1010637426934 11352461

[B4] Baldwin T. C. Hengel A. Roberts K. (2000). The C-terminal PAC domain of a secreted arabinogalactan protein from carrot defines a family of basic proline-rich proteins, in Cell and Developmental Biology of Arabinogalactan Proteins, eds Nothnagel E. A. Bacic A. Clarke A. E. (New York, NY: Kluwer Academic Publishers), 43–50 10.1007/978-1-4615-4207-0_4

[B5] Basu D. Liang Y. Liu X. Himmeldirk K. Faik A. Kieliszewski M. (2013). Functional identification of a hydroxyproline-*O*-galactosyltransferase specific for arabinogalactan protein biosynthesis in Arabidopsis. J. Biol. Chem. 288, 10132–10143 10.1074/jbc.M112.432609 23430255 PMC3617256

[B6] Battaglia M. Solorzano R. M. Hernandez M. Cuellar-Ortiz S. Garcia-Gomez B. Marquez J. (2007). Proline-rich cell wall proteins accumulate in growing regions and phloem tissue in response to water deficit in common bean seedlings. Planta 225, 1121–1133 10.1007/s00425-006-0423-9 17109151

[B7] Baumberger N. Ringli C. Keller B. (2001). The chimeric leucine-rich repeat/extensin cell wall protein LRX1 is required for root hair morphogenesis in *Arabidopsis thaliana*. Genes Dev. 15, 1128–1139 10.1101/gad.200201 11331608 PMC312681

[B8] Baumberger N. Steiner M. Ryser U. Keller B. Ringli C. (2003). Synergistic interaction of the two paralogous Arabidopsis genes *LRX1* and *LRX2* in cell wall formation during root hair development. Plant J. 35, 71–81 10.1046/j.1365-313X.2003.01784.x 12834403

[B9] Bernhardt C. Tierney M. L. (2000). Expression of AtPRP3, a proline-rich structural cell wall protein from arabidopsis, is regulated by cell-type-specific developmental pathways involved in root hair formation. Plant Physiol. 122, 705–714 10.1104/pp.122.3.705 10712533 PMC58905

[B10] Bradley D. J. Kjellbom P. Lamb C. J. (1992). Elicitor-induced and wound induced oxidative cross-linking of a proline rich plant cell wall protein: a novel, rapid defense response. Cell 70, 21–30 10.1016/0092-8674(92)90530-P 1623521

[B11] Brady J. D. Sadler I. H. Fry S. C. (1996). Di-isodityrosine, a novel tetrametric derivative of tyrosine in plant cell wall proteins: a new potential cross-link. Biochem. J. 315, 323–327 8670125 10.1042/bj3150323 PMC1217189

[B12] Brisson L. F. Tenhaken R. Lamb C. (1994). Function of oxidative cross linking of cell wall structural proteins in plant disease resistance. Plant Cell 6, 1703–1712 10.1105/tpc.6.12.1703 12244231 PMC160556

[B13] Brown D. Wightman R. Zhang Z. Gomez L. Atanassov I. Bukowski J. P. (2011). Arabidopsis genes *IRREGULAR XYLEM* (*IRX15*) and *IRX15L* encode DUF579-containing proteins that are essential for normal xylan deposition in the secondary cell wall. Plant J. 66, 401–403 10.1111/j.1365-313X.2011.04501.x 21251108

[B14] Bucher M. Brunner S. Zimmermann P. Zardi G. I. Amrhein N. Willmitzer L. (2002). The expression of an extensin-like protein correlates with cellular tip growth in tomato. Plant Physiol. 128, 911–923 10.1104/pp.010998 11891247 PMC152204

[B15] Cannon M. C. Terneus K. Hall Q. Tan L. Wang Y. Wegenhart B. L. (2008). Self-assembly of the plant cell wall requires an extensin scaffold. Proc. Natl. Acad. Sci. U.S.A. 105, 2226–2231 10.1073/pnas.0711980105 18256186 PMC2538902

[B16] Carpita N. C. Gibeaut D. M. (1993). Structural models of primary cell walls in flowering plants, consistency of molecular structure with the physical properties of the walls during growth. Plant J. 3, 1–30 10.1111/j.1365-313X.1993.tb00007.x 8401598

[B17] Cavalier D. M. Lerouxel O. Neumetzler L. Yamauchi K. Reinecke A. Freshour G. (2008). Disrupting two *Arabidopsis thaliana* xylosyltransferase genes results in plants deficient in xyloglucan, a major primary cell wall component. Plant Cell 20, 1519–1537 10.1105/tpc.108.059873 18544630 PMC2483363

[B18] Cosgrove D. J. (2005). Growth of the plant cell wall. Nat. Rev. Mol. Cell Biol. 6, 850–861 10.1038/nrm1746 16261190

[B19] Dick-Perez M. Zhang Y. Hayes J. Salazar A. Zabotina O. A. Hong M. (2011). Structure and interactions of plant cell-wall polysaccharides by two- and three-dimensional magic-angle-spinning solid-state NMR. Biochemistry 50, 989–1000 10.1021/bi101795q 21204530

[B20] Diet A. Link B. Seifert G. J. Schellenberg B. Wagner U. Pauly M. (2006). The Arabidopsis root hair cell wall formation mutant *lrx1* is suppressed by mutations in the *RHM1* gene encoding a UDP-L-rhamnose synthase. Plant Cell 18, 1630–1641 10.1105/tpc.105.038653 16766693 PMC1488927

[B21] Edens W. A. Sharling L. Cheng G. Shapira R. Kinkade J. M. Lee T. (2001). Tyrosine cross-linking of extracellular matrix is catalyzed by Duox, a multidomain oxidase/peroxidase with homology to the phagocyte oxidase subunit gp91phox. J. Cell Biol. 154, 879–891 10.1083/jcb.200103132 11514595 PMC2196470

[B22] Egelund J. Obel N. Ulvskov P. Geshi N. Pauly M. Bacic A. (2007). Molecular characterization of two *Arabidopsis thaliana* glycosyltransferase mutants, *rra1* and *rra2*, which have a reduced residual arabinose content in a polymer tightly associated with the cellulosic wall residue. Plant Mol. Biol. 64, 439–451 10.1007/s11103-007-9162-y 17401635

[B23] Ellis M. Egelund J. Schultz C. J. Bacic A. (2010). Arabinogalactan-proteins: key regulators at the cell surface? Plant Physiol. 153, 403–419 10.1104/pp.110.156000 20388666 PMC2879789

[B24] Ertl H. Hallmann A. Wenzl S. Sumper M. (1992). A novel extensin that may organize extracellular matrix biogenesis in *Volvox carteri*. EMBO J. 11, 2055–2062 1600938 10.1002/j.1460-2075.1992.tb05263.x PMC556671

[B25] Estevez J. M. Kieliszewski M. J. Khitrov N. Somerville C. (2006). Characterization of synthetic hydroxyproline-rich proteoglycans with arabinogalactan protein and extensin motifs in Arabidopsis. Plant Physiol. 142, 458–470 10.1104/pp.106.084244 16935991 PMC1586053

[B26] Ferris P. J. Woessner J. P. Waffenschmidt S. Kilz S. Drees J. Goodenough U. W. (2001). Glycosylated polyproline II rods with kinks as a structural motif in plant hydroxyproline-rich glycoproteins. Biochemistry 40, 2978–2987 10.1021/bi0023605 11258910

[B27] Frueauf J. B. Dolata M. Leykam J. F. Lloyd E. Gonzales M. Vandenbosch K. (2000). Peptides isolated from cell walls of *Medicago truncatula* nodules and uninfected root. Phytochemistry 55, 429–438 10.1016/S0031-9422(00)00336-8 11140604

[B28] Fry S. C. (2004). Primary cell wall metabolism: tracking the careers of wall polymers in living plant cells. New Phytol. 161, 641–675 10.1111/j.1469-8137.2004.00980.x 33873719

[B29] Gille S. Hansel U. Ziemann M. Pauly M. (2009). Identification of plant cell wall mutants by means of a forward chemical genetic approach using hydrolases. Proc. Natl. Acad. Sci. U.S.A. 106, 14699–14704 10.1073/pnas.0905434106 19667208 PMC2731844

[B30] Griffiths J. S. Tsai A. Y. Xue H. Voiniciuc C. Sola K. Seifert G. (2014). SALT-OVERLY SENSITIVE5 mediates Arabidopsis seed coat mucilage adherence and organization through pectins. Plant Physiol. 165, 991–1004 10.1104/pp.114.239400 24808103 PMC4081351

[B31] Guzzardi P. Genot G. Jamet E. (2004). The *Nicotiana sylvestris* extensin gene, *Ext 1.2A*, is expressed in the root transition zone and upon wounding. Biochim. Biophys. Acta 1680, 83–92 10.1016/j.bbaexp.2004.08.012 15488988

[B32] Hall Q. Cannon M. C. (2002). The cell wall hydroxyproline-rich glycoprotein RSH is essential for normal embryo development in Arabidopsis. Plant Cell 14, 1161–1172 10.1105/tpc.010477 12034904 PMC150614

[B33] Hieta R. Myllyharju J. (2002). Cloning and characterization of a low molecular weight prolyl 4-hydroxylase from *Arabidopsis thaliana*. Effective hydroxylation of proline-rich, collagen-like, and hypoxia-inducible transcription factor alpha-like peptides. J. Biol. Chem. 277, 23965–23971 10.1074/jbc.M201865200 11976332

[B34] Hijazi M. Durand J. Pichereaux C. Pont F. Jamet E. Albenne C. (2012). Characterization of the arabinogalactan protein 31 (AGP31) of *Arabidopsis thaliana*: new advances on the Hyp-*O*-glycosylation of the Pro-rich domain. J. Biol. Chem. 287, 9623–9632 10.1074/jbc.M111.247874 22270363 PMC3308734

[B35] Hijazi M. Roujol D. Nguyen-Kim H. Del Rocio Cisneros Castillo L. Saland E. Jamet E. (2014). Arabinogalactan protein 31 (AGP31), a putative network-forming protein in *Arabidopsis thaliana* cell walls? Ann Bot. [Epub ahead of print]. 10.1093/aob/mcu038 24685714 PMC4195544

[B36] Hirsinger C. Salva I. Marbach J. Durr A. Fleck J. Jamet E. (1999). The tobacco extensin gene *Ext 1.4* is expressed in cells submitted to mechanical constraints and in cells proliferating under hormone control. J. Exp. Bot. 50, 343–355

[B37] Immerzeel P. Eppinka M. M. De Vriesb S. C. Scholsa H. A. Voragen A. G. J. (2006). Carrot arabinogalactan proteins are interlinked with pectins. Physiol. Plant. 128, 18–28 10.1111/j.1399-3054.2006.00712.x

[B38] Jackson P. A. Galinha C. I. Pereira C. S. Fortunato A. Soares N. C. Amancio S. B. (2001). Rapid deposition of extensin during the elicitation of grapevine callus cultures is specifically catalyzed by a 40-kilodalton peroxidase. Plant Physiol. 127, 1065–1076 10.1104/pp.010192 11706187 PMC129276

[B39] Jauh G. Y. Lord E. M. (1996). Localization of pectins and arabinogalactan-proteins in lily (*Lilium longittorum* L.) pollen tube and style, and their possible roles in pollination. Planta 199, 251–261 10.1007/BF00196566

[B40] Keegstra K. Talmadge K. W. Bauer W. D. Albersheim P. (1973). The structure of plant cell walls: III. A model of the walls of suspension-cultured sycamore cells based on the interconnections of the macromolecular components. Plant Physiol. 51, 188–197 10.1104/pp.51.1.188 16658282 PMC367377

[B41] Keskiaho K. Hieta R. Sormunen R. Myllyharju J. (2007). *Chlamydomonas reinhardtii* has multiple prolyl 4-hydroxylases, one of which is essential for proper cell wall assembly. Plant Cell 19, 256–269 10.1105/tpc.106.042739 17220203 PMC1820956

[B42] Kieliszewski M. J. (2001). The latest hype on Hyp-*O*-glycosylation codes. Phytochemistry 57, 319–323 10.1016/S0031-9422(01)00029-2 11393510

[B43] Kieliszewski M. J. Lamport D. T. Tan L. Cannon M. C. (2011). Hydroxyproline-rich glycoproteins: form and function, in Plant Polysaccharides: Biosynthesis and Bioengineering, ed. Ulvskov P. (Oxford: Wiley-Blackwell), 321–342

[B44] Kieliszewski M. J. Leykam J. F. Lamport D. T. (1989). Trypsin cleaves lysylproline in a hydroxyproline-rich glycoprotein from *Zea mays*. Pept. Res. 2, 246–248 2520761

[B45] Kitazawa K. Tryfona T. Yoshimi Y. Hayashi Y. Kawauchi S. Antonov L. (2013). β-galactosyl Yariv reagent binds to the β-1,3-galactan of arabinogalactan proteins. Plant Physiol. 161, 1117–1126 10.1104/pp.112.211722 23296690 PMC3585584

[B46] Knoch E. Dilokpimol A. Geshi N. (2014). Arabinogalactan proteins: focus on carbohydrate active enzymes. Front. Plant Sci. 5:198 10.3389/fpls.2014.00198 24966860 PMC4052742

[B47] Koski M. K. Hieta R. Bollner C. Kivirikko K. I. Myllyharju J. Wierenga R. K. (2007). The active site of an algal prolyl 4-hydroxylase has a large structural plasticity. J. Biol. Chem. 282, 37112–37123 10.1074/jbc.M706554200 17940281

[B48] Koski M. K. Hieta R. Hirsila M. Ronka A. Myllyharju J. Wierenga R. K. (2009). The crystal structure of an algal prolyl 4-hydroxylase complexed with a proline-rich peptide reveals a novel buried tripeptide binding motif. J. Biol. Chem. 284, 25290–25301 10.1074/jbc.M109.014050 19553701 PMC2757231

[B49] Kwan J. S. Morvan H. (1995). Characterization of extracellular beta-(1,4)-xylan backbone *O*-substituted by arabinogalactans type-II in a plant-cell suspension. Carbohydr. Polym. 26, 99–107 10.1016/0144-8617(94)00098-E

[B50] Lamport D. T. (1973). Galactosylserine in extensin. Biochem. J. 133, 125–131 4721619 10.1042/bj1330125 PMC1177677

[B51] Lamport D. T. (2001). Life behind cell walls: paradigm lost, paradigm regained. Cell. Mol. Life Sci. 58, 1363–1385 10.1007/PL00000782 11693520 PMC11337279

[B52] Lamport D. T. Kieliszewski M. J. Chen Y. Cannon M. C. (2011). Role of the extensin superfamily in primary cell wall architecture. Plant Physiol. 156, 11–19 10.1104/pp.110.169011 21415277 PMC3091064

[B53] Lamport D. T. Kieliszewski M. J. Showalter A. M. (2006). Salt stress upregulates periplasmic arabinogalactan proteins: using salt stress to analyse AGP function. New Phytol. 169, 479–492 10.1111/j.1469-8137.2005.01591.x 16411951

[B54] Lamport D. T. Northcote D. H. (1960). Hydroxyproline in primary cell walls of higher plants. Nature 188, 665–666 10.1038/188665b013757790

[B55] Lamport D. T. Várnai P. (2013). Periplasmic arabinogalactan glycoproteins act as a calcium capacitor that regulates plant growth and development. New Phytol. 197, 58–64 10.1111/nph.12005 23106282

[B56] Léonard R. Petersen B. O. Himly M. Kaar W. Wopfner N. Kolarich D. (2005). Two novel types of *O*-glycans on the mugwort pollen allergen Art v 1 and their role in antibody binding. J. Biol. Chem. 280, 7932–7940 10.1074/jbc.M410407200 15591314

[B57] Léonard R. Wopfner N. Pabst M. Stadlmann J. Petersen B. O. Duus J. O. (2010). A new allergen from ragweed (*Ambrosia artemisiifolia*) with homology to Art v 1 from mugwort. J. Biol. Chem. 285, 27192–27200 10.1074/jbc.M110.127118 20576600 PMC2930718

[B58] Li Y. Q. Faleri C. Geitmann A. Zhang H. Q. Cresti M. (1995). Immunogold localization of arabinogalactan proteins, unesterified and esterified pectins in pollen grains and pollen tubes of *Nicotiana tabacum* L. Protoplasma 189, 26–36 10.1007/BF01280289

[B59] Liu C. Mehdy M. C. (2007). A nonclassical arabinogalactan protein gene highly expressed in vascular tissues, *AGP31*, is transcriptionally repressed by methyl jasmonic acid in Arabidopsis. Plant Physiol. 145, 863–874 10.1104/pp.107.102657 17885091 PMC2048811

[B60] McKenna C. Al-Assaf S. Phillips G. O. Funami T. (2006). The protein component in pectin - is it a AGP? Foods Food Ingred. J. Jpn. 211, 264–271

[B61] Merkouropoulos G. Shirsat A. H. (2003). The unusual Arabidopsis extensin gene *atExt1* is expressed throughout plant development and is induced by a variety of biotic and abiotic stresses. Planta 217, 356–366 10.1007/s00425-003-1002-y 14520562

[B62] Micheli F. (2001). Pectin methylesterases: cell wall enzymes with important roles in plant physiology. Trends Plant Sci. 6, 414–419 10.1016/S1360-1385(01)02045-3 11544130

[B63] Mohnen D. (2008). Pectin structure and biosynthesis. Curr. Opin. Plant Biol. 11, 266–277 10.1016/j.pbi.2008.03.006 18486536

[B64] Mollet J. C. Kim S. Jauh G. Y. Lord E. M. (2002). Arabinogalactan proteins, pollen tube growth, and the reversible effects of Yariv phenylglycoside. Protoplasma 219, 89–98 10.1007/s007090200009 11926071

[B65] Nguema-Ona E. Vicré-Gibouin M. Cannesan M. A. Driouich A. (2013). Arabinogalactan proteins in root-microbe interactions. Trends Plant Sci. 18, 440–449 10.1016/j.tplants.2013.03.006 23623239

[B66] Nuñez A. Fishman M. L. Fortis L. L. Cooke P. H. Hotchkiss A. T. J. (2009). Identification of extensin protein associated with sugar beet pectin. J. Agric. Food Chem. 57, 10951–10958 10.1021/jf902162t 19860469

[B67] Ogawa-Ohnishi M. Matsushita W. Matsubayashi Y. (2013). Identification of three hydroxyproline *O*-arabinosyltransferases in *Arabidopsis thaliana*. Nat. Chem. Biol. 9, 726–730 10.1038/nchembio.1351 24036508

[B68] Park S. Szumlanski A. L. Gu F. Guo F. Nielsen E. (2011). A role for CSLD3 during cell-wall synthesis in apical plasma membranes of tip-growing root-hair cells. Nat. Cell Biol. 13, 973–980 10.1038/ncb2294 21765420

[B69] Park Y. B. Cosgrove D. J. (2012). A revised architecture of primary cell walls based on biomechanical changes induced by substrate-specific endoglucanases. Plant Physiol. 158, 1933–1943 10.1104/pp.111.192880 22362871 PMC3320196

[B70] Parsons J. Altmann F. Graf M. Stadlmann J. Reski R. Decker E. L. (2013). A gene responsible for prolyl-hydroxylation of moss-produced recombinant human erythropoietin. Sci. Rep. 3:319 10.1038/srep03019 24145658 PMC3804855

[B71] Pellerin P. Pellerin P. Vidal S. Williams P. Brillouet J. M. (1995). Characterization of five type II arabinogalactan-protein fractions from red wine of increasing uronic acid content. Carbohydr. Res. 277, 135–143 10.1016/0008-6215(95)00206-9 8548786

[B72] Pena M. J. Kong Y. York W. S. O'Neill M. A. (2012). A galacturonic acid-containing xyloglucan is involved in Arabidopsis root hair tip growth. Plant Cell 24, 4511–4524 10.1105/tpc.112.103390 23175743 PMC3531849

[B73] Price N. J. Pinheiro C. Soares C. M. Ashford D. A. Ricardo C. P. Jackson P. A. (2003). A biochemical and molecular characterization of LEP1, an extensin peroxidase from lupin. J. Biol. Chem. 278, 41389–41399 10.1074/jbc.M304519200 12882982

[B74] Qi X. Y. Behrens B. X. West P. R. Mort A. J. (1995). Solubilization and partial characterization of extensin fragments from cell walls of cotton suspension-cultures, evidence for a covalent cross-link between extensin and pectin. Plant Physiol. 108, 1691–1701 10.1104/pp.108.4.1691 7659756 PMC157551

[B75] Rees D. A. Wight N. J. (1969). Molecular cohesion in plant cell walls. Methylation analysis of pectic polysaccharides from the cotyledons of white mustard. Biochem. J. 115, 431–439 5353518 10.1042/bj1150431 PMC1185121

[B76] Ringli C. (2010). The hydroxyproline-rich glycoprotein domain of the Arabidopsis LRX1 requires Tyr for function but not for insolubilization in the cell wall. Plant J. 63, 662–669 10.1111/j.1365-313X.2010.04270.x 20545889

[B77] Rose J. K. C. Lee S.-J. (2010). Straying off the highway: trafficking of secreted plant proteins and complexity in the plant cell wall proteome. Plant Physiol. Biochem. 153, 433–436 10.1104/pp.110.154872 20237018 PMC2879815

[B78] Rubinstein A. L. Marquez J. Suarez-Cervera M. Bedinger P. A. (1995). Extensin-like glycoproteins in the maize pollen tube wall. Plant Cell 7, 2211–2225 10.1105/tpc.7.12.2211 12242372 PMC161074

[B79] Saito F. Suyama A. Oka T. Yoko-O T. Matsuoka K. Jigami Y. (2014). Identification of novel peptidyl serine *O*-galactosyltransferase gene family in plants. J. Biol. Chem. 289, 20405–20420 10.1074/jbc.M114.553933 24914209 PMC4110251

[B80] Salvà I. Jamet E. (2001). Expression of the tobacco *Ext1.4* extensin gene upon mechanical constraints and localization of regulatory regions. Plant Biol. 3, 1–10 10.1055/s-2001-11746

[B81] Scheller H. V. Ulvskov P. (2010). Hemicelluloses. Annu. Rev. Plant Biol. 61, 263–289 10.1146/annurev-arplant-042809-112315 20192742

[B82] Schnabelrauch L. S. Kieliszewski M. J. Upham B. L. Alizedeh H. Lamport D. T. (1996). Isolation of pI 4.6 extensin peroxidase from tomato cell suspension cultures and identification of Val-Tyr-Lys as putative intermolecular cross-link site. Plant J. 9, 477–489 10.1046/j.1365-313X.1996.09040477.x 8624511

[B83] Seifert G. J. Roberts K. (2007). The biology of arabinogalactan proteins. Annu. Rev. Plant Biol. 58, 137–161 10.1146/annurev.arplant.58.032806.103801 17201686

[B84] Shirsat A. H. Wieczorek D. Kozbial P. (1996). A gene for *Brassica napus* extensin is differentially expressed on wounding. Plant Mol. Biol. 30, 1291–1300 10.1007/BF00019559 8704136

[B85] Showalter A. M. Keppler B. Lichtenberg J. Gu D. Welch L. R. (2010). A bioinformatics approach to the identification, classification, and analysis of hydroxyproline-rich glycoproteins. Plant Physiol. 153, 485–513 10.1104/pp.110.156554 20395450 PMC2879790

[B86] Shpak E. Barbar E. Leykam J. F. Kieliszewski M. J. (2001). Contiguous hydroxyproline residues direct hydroxyproline arabinosylation in *Nicotiana tabacum*. J. Biol. Chem. 276, 11272–11278 10.1074/jbc.M011323200 11154705

[B87] Sommer-Knudsen J. Clarke A. E. Bacic A. (1996). A galactose-rich, cell-wall glycoprotein from styles of *Nicotiana alata*. Plant J. 9, 71–83 10.1046/j.1365-313X.1996.09010071.x 8580973

[B88] Stratford S. Barne W. Hohorst D. L. Sagert J. G. Cotter R. Golubiewski A. (2001). A leucine-rich repeat region is conserved in pollen extensin-like (Pex) proteins in monocots and dicots. Plant Mol. Biol. 46, 43–56 10.1023/A:1010659425399 11437249

[B89] Tan L. Eberhard S. Pattathil S. Warder C. Glushka J. Yuan C. (2013). An Arabidopsis cell wall proteoglycan consists of pectin and arabinoxylan covalently linked to an arabinogalactan protein. Plant Cell 25, 270–287 10.1105/tpc.112.107334 23371948 PMC3584541

[B90] Tan L. Qiu F. Lamport D. T. Kieliszewski M. J. (2004). Structure of a hydroxyproline (Hyp)-arabinogalactan polysaccharide from repetitive Ala-Hyp expressed in transgenic *Nicotiana tabacum*. J. Biol. Chem. 279, 13156–13165 10.1074/jbc.M311864200 14724279

[B91] Tan L. Showalter A. M. Egelund J. Hernandez-Sanchez A. Doblin M. S. Bacic A. (2012). Arabinogalactan-proteins and the research challenges for these enigmatic plant cell surface proteoglycans. Front. Plant Sci. 3:140 10.3389/fpls.2012.00140 22754559 PMC3384089

[B92] Tan L. Varnai P. Lamport D. T. Yuan C. Xu J. Qiu F. (2010). Plant *O*-hydroxyproline arabinogalactans are composed of repeating trigalactosyl subunits with short bifurcated side chains. J. Biol. Chem. 285, 24575–24583 10.1074/jbc.M109.100149 20489210 PMC2915693

[B93] Tiainen P. Myllyharju J. Koivunen P. (2005). Characterization of a second *Arabidopsis thaliana* prolyl 4-hydroxylase with distinct substrate specificity. J. Biol. Chem. 280, 1142–1148 10.1074/jbc.M411109200 15528200

[B94] Tryfona T. Liang H. C. Kotake T. Tsumuraya Y. Stephens E. Dupree P. (2012). Structural characterization of Arabidopsis leaf arabinogalactan polysaccharides. Plant Physiol. 160, 563–666 10.1104/pp.112.202309 22891237 PMC3461546

[B95] Valentin R. Cerclier C. Geneix N. Aguie-Beghin V. Gaillard C. Ralet M. C. (2010). Elaboration of extensin-pectin thin film model of primary plant cell wall. Langmuir 26, 9891–9898 10.1021/la100265d 20222720

[B97] Velasquez S. M. Ricardi M. M. Dorosz J. G. Fernandez P. V. Nadra A. D. Pol-Fachin L. (2011). *O*-glycosylated cell wall proteins are essential in root hair growth. Science 332, 1401–1403 10.1126/science.1206657 21680836

[B99] Velasquez S. M. Salgado Salter J. Petersen B. L. Estevez J. M. (2012). Recent advances on the post-translational modifications of EXTs and their roles in plant cell walls. Front. Plant Sci. 3:93 10.3389/fpls.2012.00093 22639676 PMC3355594

[B100] Vlad F. Spano T. Vlad D. Daher F. B. Ouelhadj A. Fragkostefanakis S. (2007). Involvement of Arabidopsis prolyl 4 hydroxylases in hypoxia, anoxia and mechanical wounding. Plant Signal. Behav. 2, 368–369 10.4161/psb.2.5.4462 19704601 PMC2634214

[B101] Vlad F. Tiainen P. Owen C. Spano T. Daher F. B. Oualid F. (2010). Characterization of two carnation petal prolyl 4 hydroxylases. Physiol. Plant. 140, 199–207 10.1111/j.1399-3054.2010.01390.x 20553416

[B102] Voragen A. G. J. Coenen G. J. Verhoef R. P. Schols H. A. (2009). Pectin, a versatile polysaccharide present in plant cell walls. Struct. Chem. 20, 263–275 10.1007/s11224-009-9442-z

[B103] Wang T. Zabotina O. Hong M. (2012). Pectin-cellulose interactions in the Arabidopsis primary cell wall from two-dimensional magic-angle-spinning solid-state nuclear magnetic resonance. Biochemistry 51, 9846–9856 10.1021/bi3015532 23167456

[B104] Willats W. G. T. Knox P. Mikkelsen J. D. (2006). Pectin: new insights into an old polymer are starting to gel. Trends Food Sci. Technol. 17, 97–104 10.1016/j.tifs.2005.10.008

[B105] Wu H. De Graaf B. Mariani C. Cheung A. Y. (2001). Hydroxyproline-rich glycoproteins in plant reproductive tissues: structure, functions and regulation. Cell. Mol. Life Sci. 58, 1418–1429 10.1007/PL00000785 11693523 PMC11337276

[B106] Wu H. M. Wang H. Cheung A. Y. (1995). A pollen tube growth stimulatory glycoprotein is deglycosylated by pollen tubes and displays a glycosylation gradient in the flower. Cell 82, 395–403 10.1016/0092-8674(95)90428-X 7634329

[B107] Yuasa K. Toyooka K. Fukuda H. Matsuoka K. (2005). Membrane-anchored prolyl hydroxylase with an export signal from the endoplasmic reticulum. Plant J. 41, 81–94 10.1111/j.1365-313X.2004.02279.x 15610351

