# Supplementary material for: An update on post-translational modifications of hydroxyproline-rich glycoproteins: toward a model highlighting their contribution to plant cell wall architecture
Source: Front Plant Sci. 2014 Aug 14;5:395. doi: 10.3389/fpls.2014.00395 (PMC4132260; doi:10.3389/fpls.2014.00395)
Supplement: Supplementary file 1 [file DataSheet1.PDF]

Table A1. Enzymes involved in PTMs of EXTs. *Cr: Chlamydomonas reinhardtii*; *Dc:Daucus carota; Dca:Dianthus caryophyllus; Gv;Vitis vinifera; La:Lupines albus ;Nt: Nicotiana tabacum; Pv: Phaseolus vulgaris; Pp:Physcomitrella patents; Sl: Solanum lycopersicum; Vr: Vinca rosea; Vc: Volvox carteri.*

Gene/Protein name	*In vitro* activity	*In vivo* activity	Sub-cellular localization	Organ localization	Enzyme activity/Phenotype	Reference
**Prolyl 4-Hydroxylases (P4Hs)**						
AtP4H1 (At2g43080)	yes	-	ER, Golgi	-	Hydroxylates poly (L-Pro), synthetic peptides and $(\text{Pro-Pro-Gly})_{10}$/*AtP4H1* OX plants showed hypoxia-in-normoxia phenotype. Increased root hair length, absence of trichome, and reduction in seed size	(Hieta and Myllyharju, 2002) (Asif et al., 2009)
AtP4H2 (At3g06300)	yes	-	ER, Golgi	Root, Root hairs	-/Short root hairs	(Velasquez et al., 2011) (Tiainen et al., 2005)
AtP4H5 (At2g17720)	yes	-	ER, Golgi	Root, Root hairs	Hydroxylates EXTs preferentially. Interacts with P4H2 and P4H13/Short root hairs	Velasquez et al. (2014b)
P4H13 (At2g23096)	-	-	ER, Golgi	Root, Root hairs	-/Short root hairs	(Velasquez et al., 2011)
Cr-P4H1	yes	-	-	-	Hydroxylates Poly (L-Pro), *C. reinhardtii* HRGP Motifs, and collagen-like peptides/Crystal structure available/Defective cell wall consisting of a loose network of fibrils	(Keskiaho et al., 2007)
DcaP4H1	yes	-	-	Ovaries	Hydroxylates poly (L-Pro), synthetic Pro-rich peptides (similar to plant EXTs and AGPs)/Involved in climacteric ethylene production in cut carnation flowers	(Vlad et al., 2010)
DcaP4H2	yes	-	-	Stems, Petals	Hydroxylates poly (L-Pro)/Involved in climacteric ethylene production in cut carnation flowers	(Vlad et al., 2010)
SlP4H1, 7,9	-	-	-	Root, Root hairs	-/P4H-silenced plants had longer roots and shoots and larger leaves. Reduced levels of EXT and AGP in silenced SlP4H7, 9. Only reduced levels of AGP in silenced SlP4H1	(Fragkostefanakis et al., 2014)
VcP4H	yes	-	-	-	Hydroxylates Poly (L-Pro)/-	(Kaska et al., 1988)
PpP4H1,2,3,4,5,6a, 6b	-	-	Secretory compartmen ts	-	-/Reduced levels of Hyp on recombinant human erythropoietin in PpP4H1 mutant	(Parsons et al., 2013)
BY2-P4H	-	-	ER, Golgi	-	Hydroxylates $(\text{Pro-Pro-Gly})_{10}$/-	(Yuasa et al., 2005)
P4H	yes	-	ER	-	Hydoxylates poly (L-Pro)/ Its activity can be induced by an elicitor preparation from the fungus *Colletotrichum lindemuthianum*	(Bolwell et al., 1985)
DcP4H	yes	-	Cytoplasm	-	Hydroxylates Pro residues of protocollagen isolated from chick embryos/-	(Sadava and Chrispeels, 1971)
VrP4H	yes	-	-	-	Hydroxylates octa-L-Pro and poly (L-Pro)/-	Tanaka et al. (1980)
**Glycosyltransferases (GTs)**		-	-	-		
AtHPAT1 (At5g25265)	yes	yes	Golgi	-	-/Impaired pollen tubes growth, enhanced hypocotyl elongation, and early flowering	(Ogawa-Ohnishi et al., 2013)
AtHPAT2 (At2g25260)	yes	yes	Golgi	-	-/Impaired pollen tubes growth, enhanced hypocotyl elongation, and early flowering	(Ogawa-Ohnishi et al., 2013)
AtHPAT3 (At5g13500)	yes	yes	Golgi	-	-/Impaired pollen tubes growth, enhanced hypocotyl elongation, and early flowering	(Ogawa-Ohnishi et al., 2013)
AtRRA1 (At1g75120) AtRRA2 (At1g75110)	-	-	Golgi	-	-/Short root hairs	(Petersen et al., 2011) (Egelund et al., 2007)
AtRRA3 (At1g19360)	-	-	Golgi	-	-/Short root hairs	(Velasquez et al., 2011)
AtXEG113 (At2g35610)	-	-	Golgi	-	-/Short root hairs	(Velasquez et al., 2011)
AtSGT1 (At3g01720)	-	yes		Root, Root hairs	Ser galactosyltransferase activity/Short root hairs/Reduced levels of Gal in the mutant	(Saito et al., 2014) (Velasquez et al., 2014)
**Peroxidases (PERs)**						
AtPER3 (At1g05250)	-	-	-	-	-/Short root hairs	(Velasquez et al., 2014)
AtPER3L (At1g05240)	-	-	-	-	-/Extra long root hairs	(Velasquez et al., 2014)
AtPER8 (At1g34510)	-	-	-	-	-/Short root hairs	(Velasquez et al., 2014)
AtPER44 (At4g26010)	-	-	-	-	-/Extra long root hairs	(Velasquez et al., 2014)
AtPER73 (At5g67400)	-	-	-	-	-/Short root hairs	(Velasquez et al., 2014)
GvEP1	-	yes	-	-	Crosslinks EXTs/-	(Jackson et al., 2001)
pI 4.6 extensin PER	-	yes	-	-	Crosslinks EXTs/-	(Schnabelrauch et al., 1996)
La1 (LEP1)	-	yes	Apoplast	-	Crosslinks EXTs/-	(Price et al., 2003)

References

Asif, M., Trivedi, P., Misra, P., and Nath, P. (2009). Prolyl-4-hydroxylase (AtP4H1) mediates and mimics low oxygen response in *Arabidopsis thaliana*. *Funct Integr Genomics* 9, 525-535.

Bolwell, G., Robbins, M., and Dixon, R. (1985). Elicitor-induced prolyl hydroxylase from French bean (*Phaseolus vulgaris*). Localization, purification and properties. *Biochem J* 229, 693-699.

Egelund, J., Obel, N., Ulvskov, P., Geshi, N., Pauly, M., Bacic, A., and Petersen, B. (2007). Molecular characterization of two *Arabidopsis thaliana* glycosyltransferase mutants, *rra1* and *rra2*, which have a reduced residual arabinose content in a polymer tightly associated with the cellulosic wall residue. *Plant Mol Biol* 64, 439-451.

Fragkostefanakis, S., Sedeek, K., Raad, M., Zaki, M., and Kalaitzis, P. (2014). Virus induced gene silencing of three putative prolyl 4-hydroxylases enhances plant growth in tomato (*Solanum lycopersicum*). *Plant Mol Biol* 85, 459-471.

Hieta, R., and Myllyharju, J. (2002). Cloning and characterization of a low molecular weight prolyl 4-hydroxylase from *Arabidopsis thaliana*. Effective hydroxylation of proline-rich, collagen-like, and hypoxia-inducible transcription factor alpha-like peptides. *J Biol Chem* 277, 23965-23971.

Jackson, P., Galinha, C., Pereira, C., Fortunato, A., Soares, N., Amancio, S., and Pinto Ricardo, C. (2001). Rapid deposition of extensin during the elicitation of grapevine callus cultures is specifically catalyzed by a 40-kilodalton peroxidase. *Plant Physiol* 127, 1065-1076.

Kaska, D., Myllylä, R., Günzler, V., Gibor, A., and Kivirikko, K. (1988). Prolyl 4-hydroxylase from *Volvox carteri*. A low-Mr enzyme antigenically related to the alpha subunit of the vertebrate enzyme. *Biochem J* 256, 257-263.

Keskiaho, K., Hieta, R., Sormunen, R., and Myllyharju, J. (2007). *Chlamydomonas reinhardtii* has multiple prolyl 4-hydroxylases, one of which is essential for proper cell wall assembly. *Plant Cell* 19, 256-269.

Ogawa-Ohnishi, M., Matsushita, W., and Matsubayashi, Y. (2013). Identification of three hydroxyproline *O*-arabinosyltransferases in *Arabidopsis thaliana*. *Nat Chem Biol* 9, 726-730.

Parsons, J., Altmann, F., Graf, M., Stadlmann, J., Reski, R., and Decker, E. (2013). A gene responsible for prolyl-hydroxylation of moss-produced recombinant human erythropoietin. *Sci Rep* 3, 319.

Petersen, B., Faber, K., and Ulvskov, P. (2011). "Glycosyltransferases of the GT77 family," in *Annual Plant Reviews: Plant Polysaccharides, Biosynthesis and Bioengineering,* ed. P. Ulvskov. (Oxford, UK: Wiley-Blackwell), 305-320.

Price, N., Pinheiro, C., Soares, C., Ashford, D., Ricardo, C., and Pa, J. (2003). A biochemical and molecular characterization of LEP1, an extensin peroxidase from lupin. *J Biol Chem* 278, 41389-41399.

Sadava, D., and Chrispeels, M. (1971). Hydroxyproline biosynthesis in plant cells. Peptidyl proline hydroxylase from carrot disks. *Biochim Biophys Acta* 227, 278-287.

Saito, F., Suyama, A., Oka, T., T, Y.-O., Matsuoka, K., Jigami, Y., and Shimma, Y. (2014). Identification of novel peptidyl serine *O*-galactosyltransferase gene family in plants. *J Biol Chem* DOI. 10.1074/jbc.M114.553933.

Schnabelrauch, L.S., Kieliszewski, M.J., Upham, B.L., Alizedeh, H., and Lamport, D.T.A. (1996). Isolation of pI 4.6 extensin peroxidase from tomato cell suspension cultures and identification of Val-Tyr-Lys as putative intermolecular cross-link site. *Plant J.* 9, 477-489.

Tiainen, P., Myllyharju, J., and Koivunen, P. (2005). Characterization of a second *Arabidopsis thaliana* prolyl 4-hydroxylase with distinct substrate specificity. *J Biol Chem* 280, 1142-1148.

Velasquez, S., Poulsen, C., Oikawa, A., Ricardi, M., Marzol, E., Matsuoka, K., Scheller, H., Geshi, N., and Estevez, J. (2014). Complex regulation of Prolyl-4-hydroxylase 5 impacts root hair expansion *Plant Physiol*, in revision.

Velasquez, S., Ricardi, M., Dorosz, J., Fernandez, P., Nadra, A., Pol-Fachin, L., Egelund, J., Gille, S., Harholt, J., Ciancia, M., Verli, H., Pauly, M., Bacic, A., Olsen, C., Ulvskov, P., Petersen, B., Somerville, C., Iusem, N., and Estevez, J. (2011). *O*-glycosylated cell wall proteins are essential in root hair growth. *Science* 332, 1401-1403.

Vlad, F., Tiainen, P., Owen, C., Spano, T., Daher, F., Oualid, F., Senol, N., Vlad, D., Myllyharju, J., and Kalaitzis, P. (2010). Characterization of two carnation petal prolyl 4 hydroxylases. *Physiol Plant* 140, 199-207.

Yuasa, K., Toyooka, K., Fukuda, H., and Matsuoka, K. (2005). Membrane-anchored prolyl hydroxylase with an export signal from the endoplasmic reticulum. *Plant J* 41, 81-94.
